# Multilevel Analysis of 24-Hour Blood Pressure, Heart Rate, and Associated Factors among Police Officers in Hanoi, Vietnam

**DOI:** 10.1155/2020/7494906

**Published:** 2020-05-16

**Authors:** Dao Thi Minh An, Luu Ngoc Hoat, Dinh Thai Son, Do Thanh Toan, Luu Ngoc Minh, Phan Van Mai, Hoang Van Minh

**Affiliations:** ^1^Institute for Preventive Medicine and Public Health (IPMPH), Hanoi Medical University, 01, Ton That Tung Str, Dong Da Dist, Hanoi, Vietnam; ^2^Department of Preventive Medicine, Ministry of Public Security, 47, Pham Van Dong, Quan Hoa, Cau Giay District, Hanoi, Vietnam; ^3^Hanoi University of Public Health, 1A Duc Thang, Dong Ngac, Bac Tu Liem District, Hanoi, Vietnam

## Abstract

**Background:**

Due to long-hour outdoor working environment, policemen have been subjected to tremendous health risks including blood pressure (BP) and heart rate (HR). In tropical countries, the temperature is extremely harsh which may get peak at above 40 Celsius degrees or drops under 8 Celsius degrees. However, the existing data on the effects of weather variation on BP and HR among police task force has been scarce in Vietnam.

**Aims:**

This study aimed to describe the variation of 24-hour BP and HR and identify factors associated with BP and HR for further appropriate interventions in order to reduce health risks from occupational exposure.

**Methods:**

Multilevel regression analysis (MLRA) was applied with two levels of influent factors. 24-hour holter measured systolic blood pressure (SBP), diastolic blood pressure (DBP), and HR values were the first level which should then be nested in the second level (individual). 24-hour temperature and humidity variations were extracted, respectively, from Hanoi Hydrometeorology Department. All individual characteristics and risk behaviours were measured within 24 studying hours.

**Results:**

Temperature and humidity were major factors that influenced (74%-78%) the variation of BP and HR among the policemen population. When each of the Celsius degree temperature or percentage humidity increases, the SBP goes down by 0.44 (0.11-0.77) and by 0.2 (0.33-0.77), respectively, and the DBP goes down by 0.21 (-0.05-0.48) and by 0.12 (0.02-0.22), respectively, and vice versa. Interaction between temperature and humidity was significantly influent to SBP. The farther the time section from the first time section (0-6AM) the more the variation of the BP and HR. Transition from winter to summer made SBP and DBP decrease and vice versa. Individual characteristics including body mass index (BMI), bad life styles, and stress contributed 22% to 26% to the variation of BP and HR. Traffic policemen were at the greatest risks of the outdoor ambient variation in comparison with the firefighters and office-based policemen.

**Conclusion:**

Designing and equipping appropriate uniform and outdoor facilities could help to reduce influence of temperature and humidity variation in the outdoor workplace. Besides, training and educating programs that aimed at controlling BMI, risk behaviours, and stress for police taskforce, especially the traffic policemen, should be implemented.

## 1. Introduction

It has been well-evident that outdoor temperature negatively influences blood pressure (BP), leading to the great risks of cardiovascular diseases (CVDs) [1]. Exposure to cold temperatures can lead to vasoconstriction and tachycardia, both of which contribute to increased BP and cardiac load [2]. Exposure to hot temperature may lead to an increase in peripheral blood vessel diameter in order to lose heat by convection. This results in decreased systolic blood pressure (SBP) and diastolic blood pressure (DBP) in young and middle aged people [3]. As blood pressure decreases, to maintain correct blood perfusion necessary to the correct function of the heart/brain/kidney/liver, heart rate (HR) has to increase to compensate for this blood pressure diminution [4, 5]. In the study conducted by Xiang in Adelaide, Australia, an inverse U-shaped relationship was found between increasing of temperature and the number of workers' injury claims reported. Until the threshold temperature of up to 37.7°C, ambient temperature is positively associated with the risk of work injury for all the outdoor industries, including the construction industry, whereas a negative association was observed between the workers' injuries and temperature beyond this point [6]. Construction workers are particularly affected by heat stress, because of the body heat production caused by physically demanding tasks, and hot and humid working conditions. Field studies were conducted between August and September 2016 at two construction training grounds in Hong Kong. The study revealed that heat stress reduces construction labor productivity [7]. Workers become overheated from two primary sources: (1) the environmental conditions in which they work and (2) the internal heat generated by physical labor. Heat-related illnesses occur when the body is not able to lose enough heat to balance the heat generated by physical work and external heat sources. Weather conditions are the primary external heat sources for outdoor workers. Therefore, in the US, the Occupational Safety and Health Administration (OSAHA) developed the OSH Act in which employers have a duty to protect workers from recognized serious hazards in the workplace, including heat-related hazards. This guide helps employers and worksite supervisors prepare and implement hot weather plans. It explains how to use the heat index to determine when extra precautions are needed at a worksite to protect workers from environmental contributions to heat-related illness. Workers performing strenuous activity, workers using heavy or nonbreathable protective clothing, and workers who are new to an outdoor job need additional precautions beyond those warranted by heat index alone [8].

Among the outdoor workers, policemen are one of those having the longest outdoor working hours, especially the traffic policemen. Although various studies address the impacts of ambient occupational exposure on the variation of blood pressure and pulse, there are a limited number of studies analysing exposure to weather variation regarding SBP/DBP/HR among police officers [9–11]. Besides, previous studies also indicated factors associated with BP and HR including risk behaviours (smoking, alcohol use, and coffee drinking) [12–14] and personal factors (sex, age, types of occupation, history of hypertension, suffering from workplace, or family stress) [15–17].

Therefore, our research questions were the following: (i) to what extent the weather variation and individual characteristics contribute to the fluctuation of BP and HR among police officers? (ii) Among 3 types of policemen (traffic policemen, firemen, and office-based policemen), which one is most influenced by the variation of ambient environment?

This study aimed to describe variation of 24-hour SBP/DBP and HR and identify associated factors including temperature, humidity, and individual characteristics for further appropriate interventions.

## 2. Methods

### 2.1. Study Setting

The People's Public Security of Vietnam is the main police and security force of Vietnam, under control of the Ministry of Public Security. It is a part of the Vietnam People's Armed Forces and under the de facto control of Communist Party of Vietnam. It is composed of 2 service branches, the Vietnam People's Security Force and the Vietnam People's Police Force. Vietnam People's Police (including Civil Defence Forces) has function of working to prevent, investigate, and solve environmental, political, traffic, functional, and corruption-related criminal activities in keeping with the laws of the Socialist Republic [18, 19]. This taskforce has 7 types of jobs which are the following: police of administration and social security; police of criminal investigation; police of drug abuse investigation; police of economic investigation; police of legislation protection; police of traffic control; and police of firefighter.

The firefighter is comprehensively trained in firefighting, primarily to extinguish hazardous fires as well as to rescue people and animals from dangerous situations. Firefighters often work in emergency and dangerous working conditions such as heat exposure from fire events. Moreover, firefighters are subjected to mental health stress as results of basic tasks include the following: fire suppression, rescue, fire prevention, basic first aid, and investigations. Firefighting is further broken down into skills which include the following: size-up, extinguishing, ventilation, search and rescue, salvage, containment, mop up, and overhaul [20]. The traffic policemen are those who direct traffic or serve in a traffic or road policing unit enforcing rules of the road. Traffic police includes officers who patrol major roads and police who address traffic infractions on other roads. Thus, they are greatly exposed to the variation of weather.

Among 7 types of policemen, the firefighter and the traffic police are those that usually have most of their time working outdoors and often exposure to the variation of the weather. Within each of these two groups, there are staffs who are responsible only for administrative activities hereinafter called office-based police officers working at indoor environment. These staffs do not suffer from exposure to ambient variation.

In order to address the research question regarding how the outdoor working environment affects police taskforce, we designed to select three groups of police taskforce which are firefighter, traffic police, and office-based police officers to make comparisons.

### 2.2. Study Population, Inclusion, and Exclusion

Inclusion criteria are for all 3 types of police: any policeman working in the taskforce of firefighters, or traffic control, or administration at age of 18 and above, working at least 8 hours per working day, and willing to wear holter during 24 continuous hours in one day each in the summer and in the winter.

Specific inclusion criteria are for different types of police taskforce:

- The fire-fighters: currently trained at the Institute of Fire Control
- The traffic policemen: currently employed by the Department of Traffic Office in Hanoi
- The office based policemen: currently working as administrative staff at the Department of Traffic Office in Hanoi or the Institute of Fire Control

Exclusion criteria are for all 3 types of study subjects: eligible subjects who did not commit to wearing holter as being required or missed out any turn of wearing holter.

### 2.3. Sampling and Sample Size

300 study subjects were selected as a targeted sampling of which number of each police type was 100. To get 100 study subjects for each group, firstly, a list of a study subgroup was identified; then technique of random selection was applied by using Stata software. For those sampled but not willing to participate, other subjects from the rest of the list would be selected for replacing the refusing ones.

### 2.4. Technique for Measuring Pulse and Blood Pressure

After being selected eligibly into the study, each participant was informed about the objectives, benefit, and participants' responsibility of the study and asked to give his/her consent. Participant was asked to wear a holter for 24 continuous hours with guidance and support from trainer. He/she was also required to return on the next day for discharging the holter at the study site. Each holter, before being worn, was set to measure pulse and blood pressure every hour and to automatically back up information in its embedded memory. The day when participant returns for discharging holter, the technical assistant would transfer the backup information from the holter to the database developed and stored in a separate study computer. All information measured from the previous participant recorded in the holter was deleted before being used for the next participant. For those subjects who could not complete full 24 hours of holter wearing, they would be not eligible for being analysed. Among 300 eligible study subjects, only 244 completed their holter wearing as queries and were put into analysis.

### 2.5. Implementation for Holter Wearing in the Field

The ideology was set in light for process of identifying what the time and points in time that need to be measured are to catch the influence of the weather on outdoor working taskforce. It was considered that the summer and the winter are two seasons having large temperature amplitude during a day and seasons with extremely hot or cold days in comparison with Spring and Autumn. This study sampled the summer and winter seasons for asking study subjects to wear holter. In each specific season, we planned the days of measurement as per the following steps:

- Refer to the 3 levels of temperature, the minimum, the medium, and the maximum in summer and winter of the previous years and identify the range of each level used for making a schedule of the days for participants to wear holter
- Consult the Meteorological and Hydrological Administration about the weather forecast of a specific week one week in advance. If there are days with temperatures forecast at around any range of three levels planned, those days are sampled for asking study subject to wear holter. Each temperature level had been measured in 15 days, which made the total days for three levels in one season 45 days.

### 2.6. Study Variables

#### 2.6.1. Dependent Variable

The 24-hour diastolic blood pressure, systolic blood pressure, and pulse were measured automatically every 60 minutes by asking participants to wear holter in 24 continuous hours.

#### 2.6.2. Independent Variables

Ambient environment parameters which were temperature and humidity were measured every 60 minutes parallel with BP and pulse.

Individual characteristics included age, sex, history of hypertension, and lifestyle which includes habit of cigarette smoking, alcohol drinking, and coffee drinking, and those suffering from stress were interviewed. Weight and height were measured and converted automatically into BMI indicators. Types of occupation were checked at registration records.

### 2.7. Statistical Analysis

The multilevel regression analysis (MLRA) was applied in this study, and two levels of influent factors were developed in which the first level was the 24-hour SBP values, DBP, and pulse. Accordingly, temperature and humidity values were measured at 24 hours in the 2 seasons and variation of temperature and humidity within a day in summer and winter and interaction between temperature and humidity are considered determinants to SBP/DBP/pulse. Besides, time section variables which included 4 periods of time (6 hours/each) during a day and seasonal variables with summer and winter categories were characterized as independent variables. All these 24-hour BP and pulse values were nested within individuals created as level 2. All individual characteristics were characterized as the level 2. Lifestyle and stress were aggregated into one variable, namely, “risk.” This risk variable was the total sum of lifestyle score (smoking tobacco, alcohol drinking, and coffee drinking) and stress score (family stress and working stress). The lifestyle and the stress scores were divided into 5 levels: “never,” “rarely,” “sometimes,” “usually,” and “daily” with scores of “0,” “1,” “2,” “3,” and “4,” respectively. The total risk score ranged from “0” to “20.” The risk variable was put into the regression model. Types of occupation were divided into 3 categories based on the levels of exposing to the outdoor weather, including “none” for the office-based police officer, “sometimes” for the firemen officer, and “daily” for the traffic police officer, respectively. Gender variable was classified into female and male while history of hypertension was categorised as “yes” or “no.” Age was treated as a continuous variable.

We used multilevel regression analysis to determine factors associated with SBP/DBP/pulse in which beta coefficients (95% of confidence intervals) were identified in the fixed-effects part of the model. Three models were tested as follows.

The “empty” model or model 1 which will be used hereinafter was analysed in which there were not any explanatory variables included to see how 24-hour blood pressure differences are partitioned in a variability that exists between times of measurement from the same individual and a variability that exists between individuals.

The equation of the empty model is given as follows:

(1) $$\begin{array}{c} {SBP}_{24h}={SBP}_{SBPmean}+E_{N2c}+E_{I2c}\\ {SBP}_{24h}=SBP\;\;of\;\;24\;\;hours\;\;in\;\;an\;\;individual\\ {SBP}_{SBP\;\;mean}=Mean\;\;SBP\;\;of\;\;the\;\;study\;\;population \end{array}$$

E_N2c_ = difference between the SBP mean and the individual SBP mean (also known as individual “shrunken residual”).

E_I2c_ = difference between the individual SBP mean and the 24-hour SBP value (also known as “24 hour residual”).

The model shows that one-hour SBP of an individual (SBP_24h_) was equal to the mean SBP in the whole study population (SBP mean) plus the predicted individual difference from the SBP mean (individual shrunken residual [E_N2c_]) plus the 24-hour difference from the individual mean (that is, 24-hour residual [E_I2c_]).

In model 2, ambient variables of the first level including temperature, humidity, interaction between temperature and humidity, and variation of temperature and humidity during 24 hours were taken into account.

In model 3, apart from ambient variables using in model 2, time section and seasonal factors were also put into it.

In model 4, apart from variables using in model 2 and model 3, individual characteristics were included.

We used STATA software version 13 and MLwiN software version 3.1 to analyse the data.

It was assumed that the residuals were normally distributed and there was independence between the individual residuals and the 24-hour residuals.

**Figure 1:**
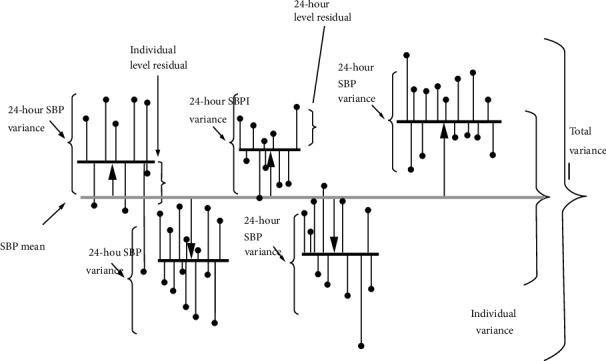
Information of the two levels used for multilevel analysis adapted from “A Brief Conceptual Tutorial of Multilevel Analysis in Social Epidemiology: Linking the Statistical Concept of Clustering to the Idea of Contextual Phenomenon,” by J. Merlo, B. Chaix, M. Yang, J. Lynch, and L. Råstam, 2005, J. Epidemiol. Community Health 59, 443.

Figure 1 presents partition of the total variance of the 24-hour systolic blood pressure (SBP) in the total study population in which the grey horizontal line represents the SBP mean of total studying sample; the thick black horizontal lines represent the individual SBP means; the black circles at the top of thin vertical lines represent the 24-hour SBP values; the length of the pillows between the SBP mean and the individual SBP means is indicated; the length of the vertical lines between the individual SBP means and the black circles at the top of the thin lines shows the 24-hour variance of SBP within each study subject.

In the MLRA, we made partition of the total variance of the 24-hour SBP in the total study population (V_Total_) into a variance that occurs in individual (V_I_) and a variance that occurs in hour (V_H_) as shown in equation (2), illustrated in Figure 1, and calculated in Table 1.

(2) $$\begin{array}{c} Vtotal=Vi+V_{H} \end{array}$$

Then we estimated the extent of influence of level 2 (individual variance) by calculating intraclass correlation (ICC) or variance partition coefficient (VPC) by using the formula in equation (3). [21].

(3) $$\begin{array}{c} ICC=\frac{Vi}{Vi+VH} \end{array}$$

## 3. Results

Table 1 demonstrates general characteristics of respondents. There were a total of 244 policemen involved in the study. There were 3 types of policemen: traffic police officer (96), office-based police officer (61), and firemen trainees (87). The office based police officers were older than the traffic police officers and the fireman trainees were the youngest with their average age range 28-46; 21-28; and 19-21, respectively. More than 90% of police officers were male except the office based police officer group with 22.9% females. None of police officers in 3 groups had BMI at the level of obesity; the traffic police officer group had BMI at level of overweight (23.2±2.5). Of note, 3.1% of the office-based officers and 1.6% of traffic police officers reported history of hypertension. In terms of life style, smoking and coffee drink were highly prevalent among the traffic police officers while alcohol drinking was commonly reported among office-based police officers. Office-based police officers reported their stress at work at the highest while the traffic police officers reported their stress at home. The firemen trainees always reported the least of all risk factors.

Table 2 presents the variation of SBP, DBP, and pulse in 4 time frames which are 1-6AM, 7-12AM, 1-6PM, and 7-12PM and between summer and winter. The sample consists of 11712 blood pressure values measured from 244 individuals working in different 3 types of policemen of which each individual was measured 24 hours one day each in summer and winter. The individual outcome variables were SBP, DBP, and pulse. In most of the cases, the SBP, DBP, and pulse varied differently in 4 time frames and between 2 seasons except the pulse of the AM section, the SBP of the 7-12PM section, and the DBP of the PM sections.

**Figure 2:**
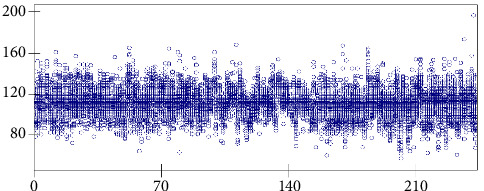
Distribution of SBP during 24 hours in summer of 244 study subjects. The vertical axis represents value of SBP in mmHg, while the horizontal axis represents the 244 individuals. The circle represents study subject's SBP measured at a particular hour during a day. Each study subject has 24 circles presented vertically.

**Figure 3:**
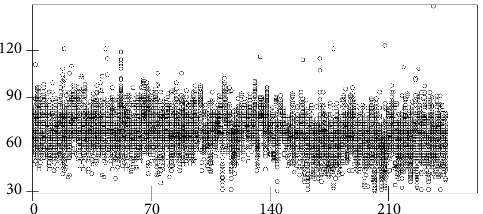
Distribution of DBP during 24 hours in winter of 244 study subjects. The vertical axis represents value of DBP in mmHg, while the horizontal axis represents the 244 individuals. The circle represents study subject's DBP measured at a particular hour during a day. Each study subject has 24 circles presented vertically.

**Figure 4:**
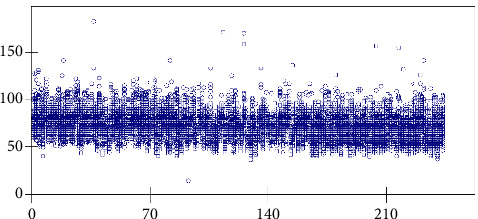
Distribution of pulse during 24 hours in winter of 244 study subjects. The vertical axis represents value of pulse rate (times per minute), while the horizontal axis represents the 244 individuals. The circle represents study subject's pulse rate measured at a particular hour during a day. Each study subject has 24 circles presented vertically.

Figure 2 illustrates the distribution of SBP during 24 hours in summer of 244 study subjects, Figure 3 presents the distribution of DBP during 24 hours in summer of 244 study subjects, and Figure 4 shows the variation of pulse during 24 hours in summer of 244 study subjects. These three figures were drawn by using Stata software and the original data of this study show that, within 24 hours of a day, the SBP, DBP, and pulse gradually increased from the first AM time section, the peak was during the working day, and they kept stable until midnight but then decreased during 1-6AM section.

Tables 3, 4, and 5 present results of the multilevel regression analysis. Ambient temperature/humidity variation and individual characteristics had significant influence on variation of the BP. The first level which was the variation of 24 ambient factors contributed most to the total variation of SBP, DBP, and pulse while the individual level contributed the least to the total variation (26%, 25%, and 23%, respectively). According to the first level, every additional unit increase in temperature and humidity or any variation of humidity during the day, or changing from winter to summer, or comparing between male and female, the SBP decrease. In contrast, if any variation of temperature during the day or any positive correlation between temperature and humidity or any transition of time section from the first section occurs or there is any additional BMI increase or any 1 risk score increase, the SBP increases, respectively. It is also the case in DBP except factor of temperature, interaction between temperature and humidity, and variation of temperature and humidity during the day and transition between summer and winter or change between male and female. There are two factors that do not get statistical significance in influence SBP but they did for DBP which are age and occupation. For pulse, only 24-hour temperature, variation of humidity during the day, transition of time section and season, age, and occupation have statistical significance contribution to the variation of the pulse. Noticeably, although risk score plays a significant role in the increase of SBP and DBP, it does not have significant influence on the pulse.

## 4. Discussion

The current study aimed to analyse to what extent the weather variation and individual characteristics contribute to the fluctuation of BP and HR among police officers and how factors within each level make the variation of BP and HR. The residual variations of level 1 are always much higher than those of level 2 that resulted in the low intraclass correlation (ICC), ranging from 22% to 26%. In other words, level 1 contributed 74% to 78% to the variation of BP and HR among policemen population. In comparison with a follow-up study on 1,831 hypertensive patients [22], we can see that weather condition contributed much more to the variation of BP among police population than that of the hypertension population. In regard to factors associated with BP and HR, when each of the Celsius degree temperature or percentage humidity increases, the SBP goes down by 0.44 (0.11-0.77) and by 0.2 (0.33-0.77), respectively, and the DBP goes down by 0.21 (-0.05-0.48) and by 0.12 (0.02-0.22), respectively, and vice versa. This association was consistent with a meta-analysis of 23 related studies which indorsed the inverse associations between ambient temperature (mean, maximum, minimum outdoor temperature and indoor temperature) and BP. Accordingly, 1°C decrease in mean daily outdoor temperature resulted in 0.26 mm Hg (95% CI: 0.2-0.3) increase in SBP and 0.13 mm Hg (95% CI: 0.1-0.2) in DBP. The increase was greater in people with conditions related to cardiovascular disease. Besides, while indoor temperature was negatively associated with SBP, its impacts on DBP were not estimated due to limited studies [23]. As such, although our subjects were not cardiology patients, they did get a greater variation of SBP and DBP than the normal ones. Moreover, several previous studies indicated that BP was not only influenced by the increasing or decreasing of ambient temperature and humidity but also influenced by the interaction between temperature and humidity when air temperature peaked to an extreme and humidity at 100% [24]. This current study showed that the SBP was influenced but not much by interaction between temperature and humidity while the DBP and pulse were not.

In addition to variation of 24-hour temperature and humidity, several studies indicated that the amplitude of individual blood pressure fluctuation with temperature in different time frames is for a year in a 29-degree centigrade range, and the variation was 9.4/7.3 mmHg [22] while this current study showed that SBP, DBP, and HR changed: 6.7 to 8.8; 5.9 to 7.5; and 9.8 to 12.3, respectively, for each time frame changed. Clearly, the farther the time section from the first time section (0-6AM) the more the variation of the BP and pulse. Moreover, transition from summer to winter or vice versa also contributed to BP and HR. Several studies showed a larger increase in the cardiovascular load in winter. This current study showed that, in any transition from winter to summer, the SBP and DBP decreased while the HR increased. Season should be taken into account in studies of blood pressure and in the diagnosis and treatment of hypertension [25].

Features of association between temperature, humidity, and cardio health should be used to design proper shift work schedules for the outdoor taskforce, especially for the traffic policemen. The shifts should be adjusted shorter when there are days with extremely cold or hot temperature in the winter and the summer as well as when there are highly humid and extremely hot days. It is also necessary to design portable outdoor working shelters/umbrellas using reflectable and convectable material to protect traffic police at mobility working sites. For individual protection, designing and offering appropriate uniforms should be taken into account. This can be achieved by asking or surveying the task force periodically to receive their ideas and suggestions.

As discussed above, although the individual characteristics played less than the ambient environment, there were factors of BMI, age occupational exposure, and risk score that had statistical significance to the variation of BP and HR among the police population.

Our study endorsed the positive association between BMI and BP. Accordingly, increasing one unit of BMI resulted in increasing 0.78 mmHg of SBP and 0.39 mmHg of DBP. Consistently, a previous study also indicated the positive correlation between BMI, fat percentage, and blood pressure in both SBP as well as DBP. Odds ratio showed that overweight/obese subjects were more likely to have hypertension than those with normal BMI [26]. It was also the case of age feature although each increase of one age year old does not make strong increase in BP. Furthermore, our study showed that the traffic police had 5.3 HR more than office-based officers while the firefighter had 3.9 mmHg of DBP less than office-based officers. While very few studies have focused on the exposure to the outdoor temperature and humidity among traffic police officers, several previous studies indicated that exposure to urban pollutants such as particulate matter, carbon monoxide [27], and benzene [15] leads to contamination [28] increasing the risks of BP. Many studies showed that people living or working near roadways were at higher risks of cardiovascular or respiratory diseases due to vehicle emissions [29].

Besides, age, occupational exposure, risk behaviours, and stress were also predictors of BP and HR among police population. A randomly selected study in England on 33.860 adults indicated that older male smokers had higher systolic BP than did nonsmoking counterparts. Among women, light smokers (1 to 9 cigarettes per day) tended to have lower BPs than heavier smokers and never smokers, significantly [13]. The previous literature showed that alcohol and caffeine intake was linked with increased SBP and DBP. It means that every cup of alcohol intake increase of 2.7 mm and 1.4 mm Hg, respectively, after alcohol intake [30] or administration of 200–300 mg caffeine produced a mean increase of 8.1 mm Hg (95% CI: 5.7, 10.6 mm Hg) in systolic BP and of 5.7 mm Hg (95% CI: 4.1, 7.4 mm Hg) in diastolic BP [31]. Stress was also a predictor of high BP. Previous studies showed that combined mental and physical stress were risk factors of high BP and HR. BP reactions to ascent that represents an accumulation of physical (mild hypobaric hypoxia) and psychological stressors depend on predetermined psychological traits (stress coping strategies) [17]. Other studies described that psychosocial stressors could cause changes in ambulatory blood pressure mean values in male police officers [32, 33].

As a result, the above-mentioned factors including smoking, alcohol/coffee intake, and stress from workplace and family were incorporated in variable, namely, “risk,” and the current study indicated that each score increase in risk made 0.5 mmHg increase in DBP.

It is important to educate the traffic police taskforce on the importance of the BMI and risk behaviours to cardiovascular conditions. Periodical education on physical exercise, healthy intake, risk behaviours, and estimation of risk scores should be planned and conducted annually to help the traffic taskforce control their BMI and reduce their risk scores.

This study should be interpreted in the light of some limitations. Among 300 study subjects, 56 did not complete their wearing holters for the whole 24 continuous hours due to the extremely hot temperature as it made the participants unpleasant. Moreover, at the beginning, the study was designed to measure extreme points of temperature in the winter and the summer to see how the BP and HR were affected. Unfortunately, some of the extreme points were not measured. In addition, ideally, the ambient environment should have been measured using a composite index, namely, “heat index,” consisting of 4 factors, surrounding temperature, humidity, speed of wind, and thermal radiation. However, this study has addressed just two out of these 4 factors, temperature and humidity. Further studies should examine the remaining two indicators to estimate an overall heat index.

## 5. Conclusions

In comparison with individual factors, ambient environment including temperature and humidity contributes majority (74%-78%) of influence to the variation of BP and HR among policemen population. When each of the Celsius degree temperature or percentage humidity increases, the SBP goes down by 0.44 (0.11-0.77) and by 0.2 (0.33-0.77), respectively, and the DBP goes down by 0.21 (-0.05-0.48) and by 0.12 (0.02-0.22), respectively, and vice versa. Interaction between temperature and humidity had significant influence on SBP. The farther the time section from the first section (0-6AM) the more the variation of the BP and pulse. Transition from winter to summer made SBP and DBP decrease and vice versa. Traffic policemen were most vulnerable to the outdoor ambient variation. To deal with these above variations of the weather to reduce their influence on the health of traffic policemen, designing and equipping with appropriate uniform and facilities may help to reduce influence of temperature and humidity variation in outdoor workplace. Besides, training and educating police taskforce in controlling their BMI, risk behaviour, and stress should be emphasized, especially for the traffic policemen group.

## Figures and Tables

**Table 1:** General characteristics of respondents (n= 244).

	Total (n = 244)	Traffic police officer (n= 96)	Office-based policemen (n = 61)	Firefighter (n = 87)
Age, median (IQR)	24.0 (21.0 - 29.0)	25.0(21.0- 28.0)	37.0 (28.0- 46.0)	20.0 (19.0- 21.0)

Gender, n (%)				

Male	222 (91.0)	90 (93.8)	47 (77.1)	85 (97.7)

Female	22 (9.0)	6 (6.2)	14 (22.9)	2 (2.3)

Height, mean (SD)	169.3 (5.1)	170.3 (4.8)	166.6 (4.8)	170.1 (4.9)

Weight, median (IQR)	63.0 (58.0 – 68.5)	64.0 (60.0- 70.0)	65.0 (60.0 - 70.0)	60.5 (57.0- 65.0)

BMI, mean (SD)	22.0 (2.3)	23.2 (2.5)	22.2 (2.3)	21.1 (1.7)

History of blood pressure				

No	240 (98.4)	60 (98.6)	93 (96.9)	87 (100.0)

Yes	4 (1.6)	1 (1.6)	3 (3.1)	0

Smoke, n (%)				

Daily	23 (9.4)	19 (19.8)	3 (4.9)	1 (1.2)

Usually	3 (1.2)	3 (3.1)	0	0

Sometimes	19 (7.8)	14 (14.6)	5 (8.2)	0

Rarely	10 (4.1)	1 (1.0)	8 (13.1)	1 (1.2)

None	189 (77.5)	59 (61.5)	45 (73.8)	85 (97.6)

Drinking, n (%)				

Daily	7 (2.9)	3 (3.1)	4 (6.6)	0

Usually	5 (2.1)	2 (2.1)	3 (4.9)	0

Sometimes	141 (57.8)	65 (67.7)	25 (41.0)	51 (58.6)

Rarely	54 (22.1)	11 (11.5)	16 (26.2)	27 (31.0)

None	37 (15.2)	15 (15.6)	13 (21.2)	9 (10.4)

Coffee, n (%)				

Daily	7 (2.9)	5 (2.1)	2 (3.3)	0

Usually	7 (2.9)	3 (3.1)	2 (3.3)	2 (2.3)

Sometimes	88 (36.1)	42 (43.8)	14 (22.9)	32 (36.8)

Rarely	68 (27.9)	11 (11.5)	20 (32.8)	37 (42.5)

None	74 (30.3)	35 (36.5)	23 (37.7)	16 (18.4)

Job Stress, n (%)				

Daily	5 (2.1)	5 (5.2)	0	0

Usually	10 (4.1)	0	1 (1.6)	9 (10.3)

Sometimes	90 (36.9)	26 (27.1)	11 (18.0)	53 (60.9)

Rarely	46 (18.8)	18 (18.7)	14 (23.0)	14 916.1)

None	93 (38.1)	47 (49.0)	35 (57.4)	11 (12.6)

Family Stress, n (%)				

Daily 4	0	0	0	0

Usually	2 (0.8)	0	1 (1.6)	1 (1.2)

Sometimes 2	25 (10.2)	0	6 (9.8)	19 (21.8)

Rarely 1	57 (23.4)	9 (9.4)	9 (14.8)	39 (44.8)

None 0	160 (65.6)	87 (90.6)	45 (73.8)	28 (32.2)

Risk, median (IQR)	6.0 (4.0 – 8.0)	7.0 (6.0 – 9.0)	4.0 (2.0 – 5.0)	7.0 (5.0 – 8.0)

**Table 2:** Distribution of SBP/DBP/pulse by time sections and 24 hours among 244 participants.

Time sections	summer		Winter		Paired T test
	Means	SD	Means	SD	
1-6 AM					

SBP	106.0	12.2	104.2	12.7	0.0000

DBP	62.9	10.0	61.3	10.5	0.0000

Pulse	63.4	12.8	63.3	12.0	0.3341

7-12 AM					

SBP	113.2	13.0	110.7	14.4	0.0000

DBP	68.8	10.3	67.4	11.6	0.0003

Pulse	73.1	13.4	73.7	13.5	0.1099

1-6 PM					

SBP	113.2	13.0	113.2	13.9	0.0000

DBP	68.8	10.3	68.7	12.1	0.4773

Pulse	73.1	13.4	77.5	14.2	0.0000

7-12 PM					

SBP	114.1	12.1	114.0	14.1	0.4339

DBP	69.6	10.4	69.8	12.3	0.3591

Pulse	75.3	12.9	76.4	13.9	0.0040

24 hours					

SBP	112.1	13.2	110.5	14.3	0.0000

DBP	67.5	10.8	66.8	12.1	0.0000

Pulse	71.8	14.1	72.7	14.6	0.0000

**Table 3:** Determinants of systolic blood pressure among policemen.

Systolic	Model 1		Model 2		Model 3		Model 4	
Fixed part intercept	*β*	95% CrI	*β*	95% CrI	*β*	95% CrI	*β*	95% CrI

Constant	111.3*∗∗∗*	[110.3,112.2]	127.7*∗∗∗*	[118.5,136.9]	120.0*∗∗∗*	[111.2,128.8]	97.4*∗∗∗*	[82.8,112.1]

Temperature			-0.4*∗*	[-0.7,-0.1]	-0.5*∗∗*	[-0.8,-0.1]	-0.4*∗∗*	[-0.8,-0.1]

Humidity			-0.3*∗∗∗*	[-0.5,-0.2]	-0.2*∗∗*	[-0.3,-0.1]	-0.2*∗∗*	[-0.3,-0.1]

Temperature*∗*Humidity			0.0*∗∗∗*	[0.0,0.0]	0.0*∗∗*	[0.0,0.0]	0.0*∗∗*	[0.0,0.0]

Variation of temperature			0.1*∗*	[0.0,0.3]	0.2*∗∗*	[0.1,0.3]	0.2*∗∗*	[0.1,0.3]

Variation of humidity			-0.0*∗*	[-0.1,-0.0]	-0.1*∗∗*	[-0.1,-0.0]	-0. 1*∗∗*	[-0.1,-0.0]

Time sections: 6 h-12h					6.8*∗∗∗*	[6.2,7.3]	6.8*∗∗∗*	[6.2,7.3]

Time sections: 13 h-18h					8.8*∗∗∗*	[8.2,9.4]	8.8*∗∗∗*	[8.2,9.4]

Time sections: 19 h-24h					8.9*∗∗∗*	[8.3,9.4]	8.9*∗∗∗*	[8.3,9.4]

Season: winter					-1.6*∗∗∗*	[-2.1,-0.9]	-1.5*∗∗∗*	[-2.1,-0.9]

Age							0.1	[-0.1,0.3]

Gender: Female							-4.6*∗∗*	[-8.1,-1.1]

Occupation: fireman							-2.4	[-6.1,1.2]

Occupation: traffic policemen							-0.9	[-3.9,2.1]

BMI							0.8*∗∗∗*	[0.3,1.2]

Hypertension history							-0.1	[-6.9,6.7]

Risk							0.6*∗∗∗*	[0.3,1.0]

Random part level 2: individual								

Variation (cons)	54.5	[44.4,64.7]	55.0	[44.8,65.3]	54.7	[44.6,64.9]	42.6	[34.6,50.6]

Random part level 1: 24 Hours								

Variation (cons)	135.8	[132.3,139.3]	134.2	[130.7,137.6]	121.1	[118.0,124.3]	121.1	[118.0,124.3]

N	11712		11712		11712		11712	

ICC	0.28		0.29		0.31		0.26	

**Table 4:** Determinants of diastolic blood pressure among policemen.

Diastolic	Model 1		Model 2		Model 3		Model 4	
Fixed part intercept	*β*	95% CrI	*β*	95% CrI	Β	95% CrI	*β*	95% CrI

Constant	67.2*∗∗∗*	[66.3,68.0]	78.1*∗∗∗*	[70. 6,85.6]	70.8*∗∗∗*	[63.6,78.0]	54.7*∗∗∗*	[42.8,66.5]

Temperature			-0.2	[-0.5,0.1]	-0.2	[-0.5,0.0]	-0.2	[-0.5,0.1]

Humidity			-0.2*∗∗∗*	[-0.3,-0.1]	-0.1*∗*	[-0.2,-0.0]	-0.1*∗*	[-0.2,-0.0]

Temperature*∗*Humidity			0.0*∗*	[0.0,0.0]	0.0*∗*	[0.0,0.0]	0.0	[-0.0,0.0]

Variation of temperature			-0.1	[-0.2,0.0]	-0.1	[-0.2,0.0]	-0.1	[-0.2,0.0]

Variation of humidity			0.0	[-0.0,0.1]	0.0	[-0.0,0.1]	0.0	[-0.0,0.0]

Time sections: 6 h-12h					5.9*∗∗∗*	[5.5,6.4]	5.9*∗∗∗*	[5.5,6.4]

Time sections: 13 h-18h					6.3*∗∗∗*	[5.9,6.8]	6.4*∗∗∗*	[5.9,6.9]

Time sections: 19 h-24h					7.5*∗∗∗*	[7.0,7.9]	7.5*∗∗∗*	[7.0,7.9]

Season: winter					-0.5*∗*	[-0.9,-0.0]	-0.4	[-0.9,0.0]

Age							0.2*∗∗*	[0.1,0.3]

Gender: Female							-2.5	[-5.3,0.3]

Occupation: fireman							-3.9*∗∗*	[-6.8,-1.0]

Occupation: traffic policemen							0.8	[-1.7,3.2]

BMI							0.4*∗*	[0.0,0.7]

Hypertension history							0.7	[-4.7,6.1]

Risk							0.5*∗∗*	[0.2,0.8]

Random part level 2: individual								

Variation (cons)	41.2	[33.5,48.8]	40.7	[33.2,48.2]	40.8	[33.3,48.3]	27.3	[22.1,32.4]

Random part level 1: 24 Hours								

Variation (cons)	90.8	[88.5,93.2]	90.1	[87.7,92.4]	81.6	[79.5,83.7]	81.6	[79.5,83.7]

N	11712		11712		11712		11712	

ICC	0.31		0.31		0.33		0.25	

**Table 5:** Determinants of heart rate among policemen.

Pulse	Model 1		Model 2		Model 3		Model 4	
Fixed part intercept	*β*	95% CrI	*β*	95% CrI	Β	95% CrI	*β*	95% CrI

Constant	72.3*∗∗∗*	[71.4,73.2]	69.8*∗∗∗*	[60.0,79.6]	52.7*∗∗∗*	[43.7,61.7]	43.4*∗∗∗*	[29.1,57.6]

Temperature			0.5*∗*	[0.1,0.8]	0.4*∗*	[0.1,0.8]	0.5*∗∗*	[0.1,0.8]

Humidity			-0.0	[-0.2,0.1]	0.0	[-0.1,0.2]	0.0	[-0.1,0.2]

Temperature*∗*Humidity			-0.0	[-0.0,0.0]	-0.0	[-0.0,0.0]	-0.0	[-0.0,0.0]

Variation of temperature			-0.1	[-0.2,0.0]	-0.1	[-0.2,0.1]	-0.1	[-0.2,0.0]

Variation of humidity			0.1*∗∗∗*	[0.0,0.1]	0.1*∗∗∗*	[0.1,0.1]	0.1*∗∗∗*	[0.1,0.1]

Time sections: 6 h-12h					9.9*∗∗∗*	[9.3,10.5]	9.9*∗∗∗*	[9.3,10.5]

Time sections: 13 h-18h					12.6*∗∗∗*	[12.0,13.2]	12.6*∗∗∗*	[12.0,13.2]

Time sections: 19 h-24h					12.3*∗∗∗*	[11.7,12.9]	12.3*∗∗∗*	[11.7,12.9]

Season: winter					2.5*∗∗∗*	[1.9,3.1]	2.5*∗∗∗*	[1.9,3.1]

Age							0.1*∗*	[0.0,0.3]

Gender: Female							1.4	[-1.9,4.6]

Occupation: fireman							-1.1	[-4.5,2.4]

Occupation: traffic policemen							5.3*∗∗∗*	[2.4,8.1]

BMI							0.1	[-0.3,0.5]

Hypertension history							-5.9	[-12.3,0.5]

Risk							-0.0	[-0.4,0.3]

Random part level 2: individual								

Variation (cons)	47.82		46.31		47.86		37.61	

Random part level 1: 24 Hours								

Variation (cons)	157.4	[153.4,161.5]	155	[151.0,159.0]	127.6	[124.3,130.9]	127.6	[124.3,130.9]

N	11712		11712		11712		11712	

ICC	0.23		0.23		0.27		0.23	

## Data Availability

The data used to support the findings of this study are available from the corresponding author upon request.
